# Radioprotective Effects on Late Third-Instar *Bactrocera dorsalis* (Diptera: Tephritidae) Larvae in Low-Oxygen Atmospheres

**DOI:** 10.3390/insects11080526

**Published:** 2020-08-12

**Authors:** Guoping Zhan, Jupeng Zhao, Fuhuan Ma, Bo Liu, Yong Zhong, Zijiao Song, Qingying Zhao, Naizhong Chen, Chen Ma

**Affiliations:** 1 Chinese Academy of Inspection and Quarantine, Beijing 100123, China; zhgp136@126.com (G.Z.); liubobj@126.com (B.L.); songzijiao0505@163.com (Z.S.); 13472252936@163.com (Q.Z.); chennz@263.net.cn (N.C.); 2 Guangzhou Customs Technology Center, Guangzhou Customs District People’s Republic of China, Guangzhou 510623, China; zhaojp@iqtc.cn; 3 Pingxiang Customs, Nanning Customs District People’s Republic of China, Pingxiang 532600, China; mfh201201@126.com (F.M.); nap.zy@hotmail.com (Y.Z.); 4 Department of Entomology, College of Plant Protection, China Agricultural University, Beijing 100193, China; 5 National Agro-Tech Extension and Service Center, Beijing 100125, China

**Keywords:** *Bactrocera dorsalis*, radioprotective effects, critical threshold, low-oxygen radiation

## Abstract

**Simple Summary:**

The oriental fruit fly *Bactrocera dorsalis* Hendel is a highly invasive fruit fly that causes extensive damage to many fruits and vegetables. Irradiation treatment is an economically effective and promising treatment measure. However, treatment efficacy is affected by the presence of low oxygen, i.e., mangoes are treated in modified atmosphere package. In order to investigate the reduced (radioprotective) effects on insects and determine its critical O_2_ level, third-instar *B*. *dorsalis* larvae were irradiated by X-rays at the doses of 8 to 64 Gy with intervals of 8 Gy. The treatments were conducted under ambient air or low-oxygen atomospheres (0%, 2%, 4%, 6%, 8% O_2_ and nitrogen). No adult emergence from treatments at 64 Gy in pure nitrogen or 56 Gy under other atmospheres, resulted in significant difference in tolerance. The results from statistical analyses indicate that differences in tolerance to radiation were significant in 0% and 2% O_2_ but insignificant in 4%, 6%, and 8% O_2_ environments when compared with radiation in ambient air. Therefore, the critical threshold is an O_2_ level of ≥4% and <6%, but a maximum radiation dose of 14 Gy can compensate for the radioprotective effects when the oriental fruit fly is treated in low-oxygen atmospheres.

**Abstract:**

Ionizing radiation creates free radicals, the effect of which is enhanced by the presence of oxygen; a low oxygen level produces radioprotective effects for insects compared with irradiation in ambient air. Modified (controlled) atmosphere packaging is used for maintaining quality and shelf-life extension; therefore, treatment efficacy may be affected, and there is a need to determine the critical O_2_ levels that may cause radioprotective effects. Late third-instar *Bactrocera dorsalis* (Hendel) larvae were irradiated in bags filled with ambient or low-oxygen air (0%, 2%, 4%, 6%, 8% O_2_) and were exposed to radiation doses of 8 to 64 Gy with intervals of 8 Gy. Efficacy was measured by the prevention of adult emergence. Dose–response data on mortality (failure of adult emergence) were analyzed via two-way ANOVA (analysis of variance), ANCOVA (analysis of covariance), and probit regression. The difference in radiotolerance was only significant in 0% O_2_ atmospheres through two-way ANOVA; therefore, the 95% confidence limits (CLs) of lethal dose ratios at LD_99_ were used to determine significant differences between treatments at different O_2_ levels. The differences in radiotolerance were significant in 0% and 2% O_2_ but insignificant in 4%, 6%, and 8% O_2_ environments when compared with radiation in ambient air. The critical threshold of radioprotective effects for late third-instar *B. dorsalis* larvae is an O_2_ level of ≥4% and <6%, but a maximum radiation dose of 14 Gy can compensate for this effect during phytosanitary irradiation treatment.

## 1. Introduction

The oriental fruit fly, *Bactrocera dorsalis* Hendel (Diptera: Tephritidae), is a highly invasive species with over 300 known commercial/edible and wild hosts. It is currently found in at least 65 countries, including most of Asia, much of the sub-Saharan countries of Africa, parts of America, Oceania, and Europe, but it is of quarantine significance to many other countries [1,2,3]. In China, the oriental fruit fly is one of the most destructive quarantine insects of tropical and subtropical fruits and vegetables, causing severe losses to citrus, guava, carambola, and mango [4,5]. Infested commodities are normally required to undergo phytosanitary treatments before export to regulated or quarantine areas [6].

Phytosanitary measures, including fumigation, temperature, and irradiation treatments, are currently used for shipped commodities, and the use of phytosanitary irradiation (PI) treatment has increased in recent years due to its advantages over other treatments; as a result, the global volume of different fresh products was approximately 30,000 tons in 2017 [3,4,7,8,9]. In order to develop treatment schedules and facilitate the application of PI treatment, many kinds of tephritid fruit fly third-instar larvae have been used for conducting radiotolerance and confirmatory studies, such as the oriental fruit fly, *B*. *dorsalis* [4,10,11,12]; the Mediterranean fruit fly, *Ceratitis capitata* Wiedemann [10,12]; the melon fly, *Zeugodacus cucurbitae* Coquillett [10,13]; and *Z. tau* Walker [14]. Currently, the International Plant Protection Convention (IPPC) is discussing draft standards related to irradiation treatment for *B*. *dorsalis*, *Z*. *tau*, and the genus *Anastrepha* to formulate international standards, as an annex to International Standard for Phytosanitary Measures (ISPM) No. 28 [15].

Fresh commodities are always allowed to be irradiated with insect-proof packing, so as to protect them from infestation, re-infestation, or contamination afterwards. Modified (controlled) atmosphere packaging (MAP) is widely used to improve the shelf life of fresh fruits by decreasing the O_2_ level (below 8%) and/or elevating the CO_2_ level (above 10%) [16,17,18]. Treatment under an O_2_ level lower than that in MAP is used as a single or combined phytosanitary treatment measure, and a draft ISPM (Draft ISPM 2014-006: Requirements for the use of modified atmosphere treatments as phytosanitary measures) is under discussion for adoption [15,19,20]. Ionizing radiation creates free radicals, the effect of which is enhanced by the presence of oxygen; thus, high oxygen tension can enhance the effects of radiation. Conversely, a low oxygen level produces radioprotective effects for insects, so radiation in low-oxygen environments is considered to be the most significant factor affecting the efficacy of PI treatment, aside from the radiation dose itself [21,22].

Although reduced effects of radiation have been validated by several studies that conducted radiation treatment in <3–5% O_2_ atmospheres, most of them performed comparisons between ambient air and pure nitrogen or MAP, whereas the difference in radiation effects was insignificant [23]. In addition, the PI treatment efficacy was not reduced when fruit flies were treated under hypoxia [12]. Therefore, more research is required to determine the critical O_2_ levels that may cause radioprotective effects for insects and at which point the efficacy of the recommended doses for commercial PI treatment may decrease when conducting treatments in low-oxygen environments [21,22,23]. Thus, the objective of this research was to investigate the critical O_2_ level (threshold value) that may induce radioprotective effects for late third-instar *B. dorsalis* larvae and to estimate the radiation dose that compensates for this reduced efficacy.

## 2. Materials and Methods

### 2.1. Insect Rearing

The insects used in this research were originally collected in a mango orchard in Chongzuo city, Guangxi Zhuang Autonomous Region, China in July 2018, and then reared in the Laboratory of Phytosanitary Treatment and Equipment, Chinese Academy of Inspection and Quarantine in Beijing, China. Late third-instar *B*. *dorsalis* larvae that emerged from mango fruits were collected and transferred to plastic boxes containing moist sterile sand for pupation. About 10 days later, the pupae were placed in the rearing cages (40 × 40 × 50 cm) for adult emergence; the adults were fed with sterile water, fresh fruit pulp, and a solid mixture of sucrose and hydrolyzed yeast (3:1). Eggs were collected by placing papaya peel domes (emptied of fruit pulp) in the cages, and larvae were reared on artificial diets, the components of which were described by Liu et al. [24]. The rearing room was controlled at a temperature of 24–26 °C with 50–70% relative humidity and a photoperiod of 14:10 h (L/D). The emerged late third-instar larvae (about 8 days old) in the third to sixth generations were used for conducting all the radiation treatments.

### 2.2. Handling of Larvae

Two-liter gastight air bags, purchased from Dalian Delin Gas Packaging Co., Ltd. Dalian, China, were used for conducting radiation treatments in hypoxia or ambient air. For each treatment, about 100 late third-instar larvae were wrapped in a small net bag and placed into an air bag through its opening, followed by sealing of the bag, exhausting all the air with a one-liter air-tight syringe, and injecting pure nitrogen (<0.001% O_2_) or controlled atmosphere (CA) (2%, 4%, 6%, or 8% O_2_ and nitrogen) (Beijing Green Oxygen Tiangang Technology Development Co., Ltd. Beijing, China) into the bag and holding it for one minute. The procedure was repeated three times to purify the gas in the air bag; the bags containing larvae and a volume of 0.7–0.8 L CA were subjected to irradiation treatment 15 min later. For irradiation treatment in ambient air, the larvae were wrapped and placed in the air bags without any additional procedure.

### 2.3. Radiation Treatments

All the radiation treatments were conducted in an RS-2000 Pro X-ray irradiator (Rad Source Technologies, Inc., Coral Springs, FL, USA) with operating parameters of 220 KV and 17.6 mA. In order to get a good dose uniformity ratio (1.05), the irradiator was equipped with a reflector placed in the bottom of the exposure chamber (width, 17 inch; depth, 15 inch; height, 17 inch) [25]. The bag containing larvae was placed in the exposure chamber to be irradiated at doses of 0 (control) to 64 Gy with intervals of 8 Gy. For each dose treatment, three bags (as replicates) were placed in the exposure chamber and irradiated at the same time. To measure and control the applied radiation doses, a RadCal dosimeter (model 2086, RadCal Corp., Monrovia, CA, USA) with a 10 × 6-0.6 Ion Chamber was placed near the insects. After each radiation treatment run, the cumulative dose was recorded from the dosimeter, and the irradiator was stopped when the cumulative dose was reached. The dose rate monitored in the study was about 5.0 Gy/min.

After treatment, all the bags were opened when the total sealing time reached about one hour; then, the larvae were removed and placed in sterile moist sand for pupation. Three weeks later, the numbers of late third-instar larvae, pupae, and adults were counted.

### 2.4. Data Analyses

Dose−response data on mortality (failure of adult emergence) were adjusted by using Abbott’s formula [26] and then subjected to two-way ANOVA (analysis of variance) to analyze the individual effects of radiation dose and PO_2_ (O_2_ level: 0%, 2%, 4%, 6%, 8%, and 21%) and the interaction effects of dose × PO_2_; means were compared by Tukey’s multiple comparison tests [27]. Linear regression after analysis of covariance (ANCOVA) was also used to analyze the mortality data to compare the radiotolerance of *B*. *dorsalis* treated in different O_2_ atmospheres; all the data less than 100% and the lowest dose causing 100% mortality were used in the analyses [13,27]. All the data derived from treatments below 56 Gy and from the control were subjected to probit analyses using the PoloPlus 2.0 program to estimate the minimum dose for preventing adult emergence at each environmental O_2_ level via probit and logit models (using the non-transformed dose) [28]. In addition, to compare the significance of radiotolerance between treatments at different O_2_ levels, pairwise comparison tests were performed by calculating the 95% confidence limits (CLs) of the lethal dose ratios at LD_99_ (the minimum lethal dose leading to 99% mortality at a specific confidence level, i.e., 95%) [29]. If the 95% CL excludes 1, then the LD_99_ values are significantly different [28,30].

## 3. Results

### 3.1. Effects of Radiation Dose and Oxygen Level

The percent mortality of late third-instar *B. dorsalis* larvae treated under low oxygen and ambient air were found to be significantly affected by radiation doses (*F*_7,143_ = 1089.0, *p* < 0.0001), PO_2_ (*F*_5,143_ = 4.6, *p* = 0.0026), and dose × PO_2_ interactions (*F*_35,143_ = 1.7, *p* = 0.0255) when the dose−response data were analyzed by two-way ANOVA (Table 1). With increasing radiation dose, adult emergence declined across all PO_2_ treatment groups, and no insects successfully emerged as adults when treated at 64 Gy in pure nitrogen (<0.001% O_2_) or at 56 Gy in other environments (2%, 4%, 6%, 8%, and 21% O_2_). For treatments in pure nitrogen, the mean mortality at all doses (65.0 ± 31.6%), as well as that at 56 Gy (95.5 ± 2.3%), was significantly lower than those under other treatments, suggesting that the radiation effect is decreased significantly under extreme hypoxia. Meanwhile, other statistical methods were needed to compare the significance in radiotolerance between treatments in ambient air and low-oxygen atmospheres.

### 3.2. Estimating Doses for the Prevention of Adult Emergence

#### 3.2.1. Linear Regression

**Figure 1:**
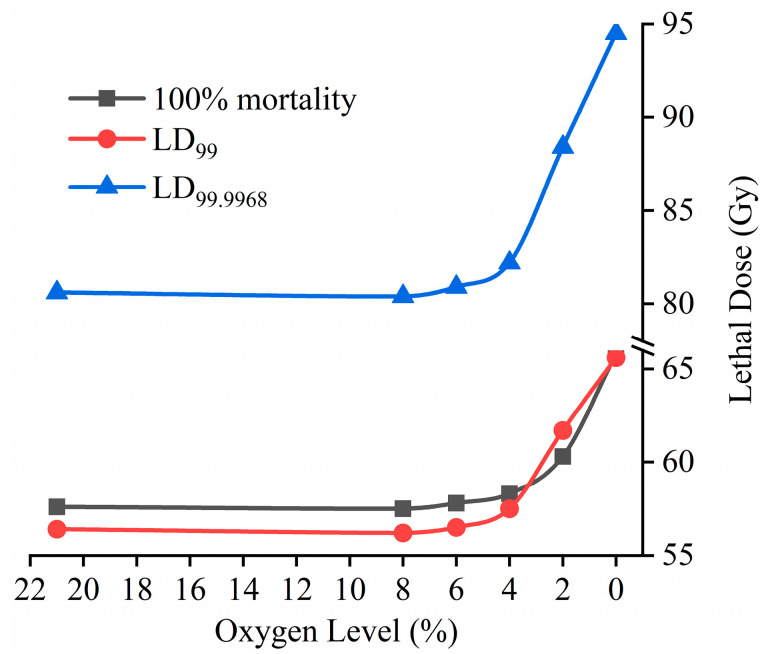
The estimated radiation dose for 100% mortality, LD_99_, and LD_99.9968_ to prevent adult emergence from late third-instar *Bactrocera dorsalis* larvae irradiated in ambient air and 0%, 2%, 4%, 6%, and 8% O_2_ environments.

The results obtained from ANCOVA and linear regression (Table 2) showed that all the coefficient of determination (*R^2^*) values were larger than 0.95 (maximum of 0.985), which implies that the regression fit the data well. As the interaction effect between dose and PO_2_ was significant (*F*_5,114_ = 2.77, *p* = 0.0212), the minimum dose for 100% mortality was predicted by liner regression to compare the relative radiotolerance among treatments in a low-oxygen atmosphere and in ambient air. The estimated dose leading to 100% mortality decreased gradually with the increasing O_2_ level from 0% to 4%, and then remained essentially unchanged (Table 2, Figure 1), suggesting that an O_2_ level of 4% is likely to be the critical threshold of radioprotective effects for late third-instar *B. dorsalis* larvae. Furthermore, compared with that in ambient air, the minimum radiation doses for 100% mortality increased from 57.6 (in ambient air) to 58.3 (1.2%), 60.3 (4.7%), and 65.8 Gy (14.2%) in 4%, 2%, and 0% O_2_ atmospheres, respectively.

#### 3.2.2. Probit Analyses

**Figure 2:**
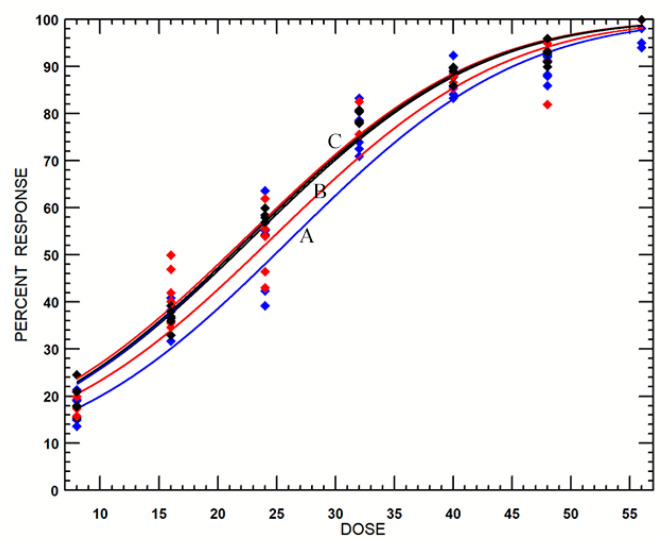
The estimated mortality curves derived from probit analyses of the dose–mortality data when late third-instar *Bactrocera dorsalis* larvae were irradiated in ambient air and in 0%, 2%, 4%, 6%, and 8% O_2_ environments. (A: 0% O_2_; B: 2% O_2_; C: 4%, 6%, 8%, and 21% O_2_).

Probit analyses were used to analyze the dose–mortality data of late third-instar *B. dorsalis* larvae. The estimated values of LD_99_ and LD_99.9968_ (the minimum lethal dose achieving a mortality of 99.9968% at a specific confidence level, i.e., 95%) analyzed by the probit and logit model were very close; thus, only the parameters derived from the probit model, such as slope, intercept, heterogeneity, estimated lethal doses, and their 95% CLs, are listed in Table 3. A small heterogeneity factor and a narrow LD_99_ confidence interval clearly showed that the analysis fit the data well. The slopes increased but the estimated values of LD_99_ and LD_99.9968_ decreased sharply with increasing the O_2_ level from 0% to 4% and were almost unchanged until 21% (Table 3, Figure 1). Similarly, the mortality curves obtained from PoloPlus software (Figure 2) can be divided into three groups, in which curve B (2% O_2_) is above curve A (0% O_2_) but below the merged curve clusters C (4%, 6%, 8%, and 21% O_2_). Furthermore, the 95% CLs of the lethal dose ratios at LD_99_ were pairwise compared between treatments at all O_2_ levels; the results (Table 3) showed that the radiotolerance of *B. dorsalis* irradiated in the 0% and 2% O_2_ environments was significantly greater than that of others, but it was insignificant when irradiated in 4%, 6%, or 8% O_2_ or in ambient air. Similar tendencies were obtained by comparing the estimated values of LD_99.9968_, though they were extrapolative values (Table 3, Figure 1). Thus, the sequence of radiotolerance is suggested as follows: 0% > 2% > 4% ≈ 6% ≈ 8% ≈ 21% O_2_ atmospheres.

Compared with that in ambient air, the LD_99.9968_ value was increased by 13.9 (17.2%), 8.0 (10.0%), and 1.8 Gy (2.2%) when larvae were irradiated in 0%, 2%, and 4% O_2_ atmospheres, respectively. Therefore, the additional radiation dose that might compensate for the radioprotective effects is less than 13.9 Gy (nominally 14 Gy) when the oriental fruit fly is treated in low-oxygen atmospheres.

## 4. Discussion

Radiotolerance in insects develops with their age and developmental time, so the most developed stage is the most radiotolerant when a common measure of efficacy is used [21]. The late third instar has been defined as the most tolerant stage of *B. dorsalis* larvae, as well as of other tephritid fruit flies, and was selected as the target stage for conducting dose–response tests in our research [4,10,11,12,13,14]. The third-instar larvae were irradiated in a series of O_2_ levels with the same dose to compare the relative tolerance between treatments, and the prevention of adult emergence was selected as the common efficacy criterions [4,9,11,31,32]. Radiation tolerance in insects is modified by the oxygen level and increases in low-oxygen atmospheres; in particular, insects irradiated in very low oxygen (<1% O_2_) have repeatedly been shown to have an increased radioprotective response [10,23]. In our study, an increased radiotolerance of *B*. *dorsalis* in low-oxygen environments (0%, 2%, and 4% O_2_) was confirmed by comparing mortality rates using two-way ANOVA (Table 1), the estimated minimum doses for 100% mortality by linear regression after ANCOVA (Table 2, Figure 1), and LD_99_ by probit analysis (Table 3, Figure 1 and Figure 2). The results are concordant with those from other radiation treatments in low-oxygen atmospheres against the Caribbean fruit fly, *Anastrepha suspensa* Loew [33]; *C. capitata* [34]; *Z. cucurbitae* [13]; *Drosophila suzukii* Matsumura [35]; the Oriental fruit moth, *Grapholita molesta* Busck [36]; the European corn borer, *Ostrinia nubilalis* Hübner [37]; and the cabbage looper moth, *Trichoplusia ni* Hübner [22].

Compared to those for radiation in ambient air, the minimum dose values for 100% mortality, LD_99_, and LD_99.9968_ estimated in pure nitrogen were increased by 8.2 (14.2%), 9.2 (16.2%), and 13.9 Gy (17.2%), respectively (Table 2 and Table 3). The estimated LD_99_ in ambient air in our research (56.4 (53.9–59.3) Gy) (Table 3) is very close to that reported by Srimartpirom et al. (58.11 (53.63–64.46) Gy), indicating that the experiments should be replicated and statistically analyzed to ensure that data are verifiable and reproducible [11,38]. The estimated LD_99.9968_ values can be used as the minimum dose for PI treatment if they are validated by large-scale confirmatory tests [32,38], and our LD_99.9968_ data (80.6 (76.3–85.6) Gy) (Table 3) are very close to the values (84.1 (73.6–99.3) Gy) that were estimated and validated by Zhao et al. [4]; thus, a maximum radiation dose of 14 Gy can compensate for radioprotective effects during the PI treatment of oriental fruit fly. This explains why no radioprotective effects were observed when a radiation dose of 116 Gy (31.9 to 35.4 Gy higher than the LD_99.9968_ estimates) was applied to late third-instar *B. dorsalis* larvae under severe hypoxia (0.3 ± 0.02% O_2_, 21.6 ± 0.1% CO_2_) [12].

Commercial PI treatment in MAP or controlled atmospheres is more complicated [39]. The minimum applied dose should be equal to or higher than the maximum dose in the confirmatory tests; therefore, it is much greater than the LD_99.9968_ estimates, and radioprotective effects are not observed as a result [12,21,31]. Moreover, although the treatment efficacy is reduced by low-oxygen atmospheres, it is increased by the presence of CO_2_, low temperature, and the long duration of modified atmosphere treatment after radiation treatment [11,12,32,38].

In order to define the critical threshold of radioprotective effects, radiation treatments in a series of oxygen levels should be carried out and the dose–response data subjected to statistical analysis to determine the statistically significant differences in radiotolerance [22,23]. The probit model is commonly used to analyze dose–response data [38,40]; however, the values of the slope obtained in this research (Table 3) are unequal, which means that the regression lines are unparallel, whereas they were parallel when Srimartpirom et al. treated *B. dorsalis* in MAP [11]. Therefore, the 95% CLs of the lethal dose ratios at LD_99_ (ratio test) were used for comparing the relative radiotolerance [28,39]. As a result, oriental fruit fly irradiated in 0% and 2% O_2_ showed significantly higher tolerance than that irradiated in ambient air and other low-oxygen atmospheres (Table 3, Figure 1 and Figure 2). There were no differences among 6% and 8% O_2_ and ambient air; however, visible but insignificant tolerance differences were present between the treatments in 4% and 6% O_2_ (Table 2 and Table 3; Figure 1 and Figure 2). To analyze the trends more intuitively, like how Chao et al. [23] predicted the critical O_2_ level for the radiation treatment of *T. ni*, the estimated values (100% mortality, LD_99_, and LD_99.9968_) at all O_2_ levels in our testing were plotted in Figure 1. All three curves remained flat through the treatment at 21%, 8%, and 6% O_2_ but turned up slowly for treatments at 6–4% O_2_ and then steepened rapidly; therefore, the critical threshold of radioprotective effects is an O_2_ level of ≥4% and <6% when late third-instar *B. dorsalis* larvae are irradiated in low-oxygen atmospheres. Since there is a small difference in LD_99_ values of 1.1 Gy (1.95% of the radiation doses) between the treatments in 4% and 6% O_2_ (Table 3), more precise research is required to reduce the range of critical values, for example, by conducting radiation testing in 4.5% or 5% O_2_ atmospheres. Similarly, radioprotective effects have been observed in other fruit insects irradiated at <3–5% O_2_ levels, such as *A. suspensa* [33], *C. capitata* [34], *Z. cucurbitae* [13], *D. suzukii* [35], *G. molesta* [36], *O. nubilalis* [37], and *T. ni* [22,23]. Furthermore, all these results support the APHIS’ (Animal and Plant Health Inspection Service, United States Department of Agriculture) changes to administrative requirements, wherein the minimum O_2_ level was reduced from 18% to 10% for conducting PI treatments [22,41]. However, the IPPC prohibits the use of PI treatment under low-oxygen atmospheres except for the treatment of *G. molesta* [20]; therefore, more insect species in different orders or families still need to be irradiated under low-oxygen environments to determine their critical threshold of radioprotective effects or the extra radiation dose that can compensate for the reduced efficacy. After that, a generic threshold of radioprotective effects can be established to facilitate the application of PI treatment of commodities with MAP or in low-oxygen environments.

Probit analysis has been widely used to analyze dose–response data for a number of fruit fly studies, and the ratios test is often used to determine the relative toxicity among a number of chemicals to determine the relative susceptibility of populations to pesticide resistance [4,11,14,29,42,43,44]. In addition, confidence interval (CI) overlap, relative median potency, and one-way ANOVA (on LD_95_) have been used to determine the significance of radiotolerance differences [11,23,30]. The 95% CLs of the lethal dose ratios at a specific efficacy level, which are calculated automatically using computer software, are more general than the alternative statistics: Relative potency, which assumes that the regression lines are parallel; the CI overlap test, which should be used only when no alternative test exists; and one-way ANOVA, which requires at least three replicates of the LD_X_ (for example, four replicates were conducted in radiation treatment of the cabbage looper moth, resulting in significant radioprotective effects determined in <0.1% and 2.5% O_2_ atmospheres) [11,23,40]. The ratio test showed that the LD_99_ value in 2% O_2_ atmospheres is significantly larger than those in air and in 4%, 6%, and 8% atmospheres (Table 3), but all the 95% CIs overlapped, suggesting that the ratio test is more sensitive than the CI overlap test, which provided a lower observed type I error rate of 0.004 to 0.005 [30]. Since probit-9 (LD_99.9968_ at 95% CL), which is well known for use as the criterion for the PI treatment of insects, is an extrapolated value, the LD_99_ that is calculated from the dose–response curve of tests in the most tolerant life stage is used as the minimum treatment level [38]; therefore, the 95% CLs of the lethal dose ratios at LD_99_ are recommend for comparing the significance of tolerance in phytosanitary treatments.

## 5. Conclusions

In this study, radioprotective effects were demonstrated and their critical threshold was determined by irradiating late third-instar *B. dorsalis* larvae with 220 KV X-rays in ambient air and low-oxygen environments. The ratio test indicated that the differences in radiotolerance were significant in 0% and 2% O_2_ but insignificant in 4%, 6%, and 8% O_2_ environments when compared with treatment in ambient air; the critical threshold of radioprotective effects is, therefore, an O_2_ level of ≥4% and <6%, but a maximum radiation dose of 14 Gy can compensate for this effect during PI treatment. We recommend that the 95% CLs of the lethal dose ratios at LD_99_ be used for comparing the significance of tolerance in phytosanitary treatments, and a generic threshold of radioprotective effects should be established by testing a variety of insects.

## Figures and Tables

**Table 1:** Radiation effects on the prevention of adult emergence as a result of irradiating late third-instar *Bactrocera dorsalis* larvae with X-rays (0–64 Gy) under ambient air and low-oxygen atmospheres.

O_2_ (%)	% Mortality at the Specified Radiation Dose (Mean ± SD)								
	0 Gy	8 Gy	16 Gy	24 Gy	32 Gy	40 Gy	48 Gy	56 Gy	64 Gy
0	6.8 ± 2.1a *	10.4 ± 2.6a	32.3 ± 5.1a	41.3 ± 8.6a	70.5 ± 1.6b	83.2 ± 1.1a	86.5 ± 1.4a	95.5 ± 2.3b	100.0 ± 0a
2	8.0 ± 1.8a	12.2 ± 1.9a	36.9 ± 8.0a	43.5 ± 6.3a	78.5 ± 1.8a	85.5 ± 1.4a	87.6 ± 7.2a	100.0 ± 0a	100.0 ± 0a
4	7.7 ± 3.2a	13.2 ± 4.9a	31.0 ± 3.4a	52.3 ± 2.4a	78.1 ± 1.3a	86.9 ± 2.1a	91.1 ± 1.8a	100.0 ± 0a	100.0 ± 0a
6	7.0 ± 0.8a	11.9 ± 4.3a	32.1 ± 1.2a	55.5 ± 4.8a	78.5 ± 3.1a	88.5 ± 3.6a	91.4 ± 0.8a	100.0 ± 0a	100.0 ± 0a
8	7.9 ± 0.4a	10.9 ± 2.1a	36.0 ± 6.6a	53.8 ± 4.5a	76.9 ± 3.8ab	87.2 ± 0.3a	93.5 ± 2.9a	100.0 ± 0a	100.0 ± 0a
21	7.9 ± 1.6a	12.0 ± 1.9a	31.5 ± 1.1a	54.8 ± 1.7a	77.2 ± 1.7a	87.3 ± 2.2a	92.8 ± 2.7a	100.0 ± 0a	100.0 ± 0a

* Within a column, means followed by the same letter are not significantly different at *p* < 0.05, using the Tukey test.

**Table 2:** Linear regressions of data on the prevention of adult emergence from late third-instar *Bactrocera dorsalis* larvae irradiated with X-rays under ambient air and low-oxygen atmospheres.

O_2_ (%)	Slope ± SE	Intercept ± SE	*R^2^*	Estimated Dose for 100% Mortality (Gy)
0	1.454 ± 0.047	−5.640 ± 1.912	0.9772	65.8
2	1.618 ± 0.091	−7.568 ± 3.313	0.9517	60.3
4	1.658 ± 0.059	−6.668 ± 2.094	0.9769	58.3
6	1.665 ± 0.061	−6.194 ± 2.198	0.9748	57.8
8	1.679 ± 0.048	−6.505 ± 1.728	0.9845	57.5
21	1.683 ± 0.047	−6.970 ± 1.673	0.9855	57.6

**Table 3:** Probit analysis on the prevention of adult emergence from late third-instar *Bactrocera dorsalis* larvae irradiated with X-rays in ambient air and low-oxygen atmospheres.

O_2_ (%)	No. Treated	Slope ± SE	Intercept ± SE	Estimated Lethal Dose (95% CLs) (Gy) *		Hetero- Geneity
				LD_99_	LD_99.9968_	
0	2155	0.058 ± 0.003	−1.486 ± 0.09	65.6 (61.7–70.5)a	94.5 (87.7–103.0)a	1.61
2	2217	0.063 ± 0.003	−1.540 ± 0.099	61.7 (56.6–68.6)a	88.4 (79.7–100.5)ab	3.13
4	2289	0.068 ± 0.003	−1.557 ± 0.096	57.5 (54.4–61.2)b	82.2 (76.9–88.8)b	1.44
6	2335	0.069 ± 0.003	−1.553 ± 0.092	56.5 (53.3–60.5)b	80.9 (75.3–87.9)b	1.76
8	2142	0.069 ± 0.003	−1.579 ± 0.100	56.2 (53.3–59.8)b	80.4 (75.2–86.7)b	1.32
21	2103	0.069 ± 0.003	−1.558 ± 0.100	56.4 (53.9–59.3)b	80.6 (76.3–85.6)b	0.99

* Within each column, values followed by different letters were significantly different based on lethal dose ratio tests (*p* < 0.05).
